# Metabolome‐Wide Mendelian Randomization Identifies Maleate as a Mediator of the Effect of Obesity on the Risk of Small Cell Lung Cancer

**DOI:** 10.1002/fsn3.70918

**Published:** 2025-11-18

**Authors:** Kui Li, Zhaodi Yang, Haifei Li, Cheng Yan

**Affiliations:** ^1^ Department of Pharmacy Xinxiang Central Hospital Xin Xiang China; ^2^ School of Pharmacy, Key Laboratory of Nano‐Carbon Modified Film Technology of Henan Province Xinxiang University Xinxiang China

**Keywords:** maleate, MR, obesity, SCLC

## Abstract

Obesity is a well‐established risk factor for numerous types of cancer, including small cell lung cancer (SCLC). However, the underlying mechanisms remain largely unclear. This research explores the causal relationships between obesity, circulating metabolites, and the risk of SCLC, aiming to identify potential metabolic intermediaries. To achieve this, a two‐step Mendelian randomization (MR) approach was employed to examine metabolites mediating the effect of obesity on the risk of SCLC. In Step 1, MR identified metabolites causally associated with SCLC, confirmed with an independent SCLC genome‐wide association study (GWAS) as the outcome. In Step 2, whole‐body fat mass was examined as the exposure to assess its causal effects on the metabolites identified in Step 1, with further validation using body mass index (BMI) as an alternative exposure. Sensitivity analyses confirmed robust causal inference. The product of coefficients approach for testing mediation quantified the role of specific metabolites in linking obesity to the risk of SCLC. In the initial screening, 1400 circulating metabolites were tested for their association between obesity and the risk of SCLC, and 55 metabolites with significant causal associations were identified. Subsequent MR analyses showed that whole‐body fat mass had an effect on 12 of these metabolites, and maleate levels were associated with both obesity and increased SCLC risk. Validation using BMI as an alternative exposure confirmed the causal association between obesity and maleate levels. Further validation using independent GWAS datasets for SCLC confirmed the causal association between maleate levels and the risk of SCLC. Mediation analysis revealed that maleate partially mediated the relationship between obesity and the risk of SCLC, accounting for 14.9% of the effect when using whole‐body fat mass as the exposure and 5.23% when using BMI as the exposure. This study highlights maleate as a key metabolic mediator in the obesity–SCLC pathway, which may offer novel insights into the metabolic mechanisms underlying the increased risk of obesity‐related cancer.

## Graphical abstract

A Two‐Step Mendelian randomization (TSMR) framework identified maleate as a circulating metabolite that mediates the causal pathway from obesity to the risk of small cell lung cancer (SCLC). Whole‐body fat mass and Body Mass Index (BMI) showed causal effects on circulating maleate levels, which in turn were associated with increased SCLC risk. Mediation analysis revealed that maleate partially explained the effect of obesity on the risk of SCLC, highlighting a potential metabolic target for intervention.

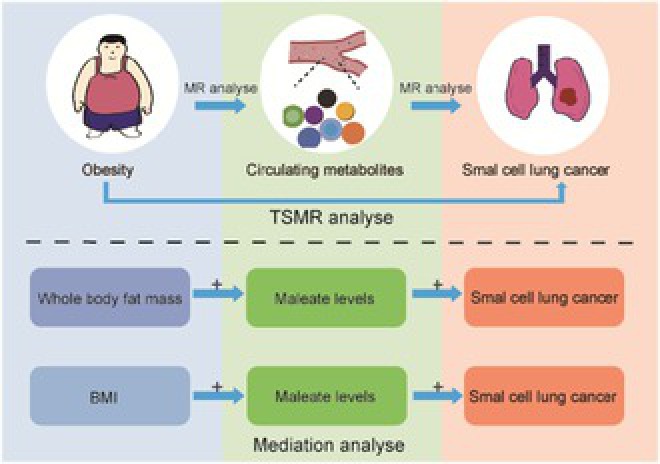

**Abbreviations**
BMI: Body Mass Index
CLSA: Canadian Longitudinal Study on Aging
GIANT: Genetic Investigation of Anthropometric Traits
GWAS: Genome‐Wide Association Studies
ILCCO: International Lung Cancer Consortium
IVs: Instrumental Variables
IVW: Inverse‐Variance Weighted
LC3: lung Cancer Cohort Consortium
LD: Linkage Disequilibrium
ME: MR‐Egger
MR: Mendelian Randomization
MWMR: Metabolome‐Wide Mendelian Randomization
ORs: Odds Ratios
SCLC: Small Cell Lung Cancer
SM: Simple Mode
SNPs: Single Nucleotide Polymorphisms
TRICL: Transdisciplinary Research for Cancer in Lung
UKB: UK Biobank
WMED: Weighted Median
WMOD: Weighted Mode

## 1 Introduction

Small cell lung cancer (SCLC) is a high‐grade neuroendocrine carcinoma and accounts for approximately 15% of all lung cancer cases worldwide (Megyesfalvi et al. 2023; Rudin et al. 2021). Despite representing a smaller subset of lung cancers, SCLC is still a major clinical challenge due to its aggressive nature and poor prognosis. It is estimated that approximately 250,000 new cases are diagnosed annually worldwide and more than 200,000 individuals die of this malignancy (Wang et al. 2023). The 5‐year survival rate remains less than 7%, highlighting the lack of substantial progress in effective therapeutic options over recent decades (George et al. 2024). The lethality of SCLC stems from distinct biological characteristics, including rapid growth, high vascularity, and profound imbalances in apoptotic regulation, all of which drive its treatment resistance and aggressive spread. Furthermore, the propensity for early and extensive metastasis exacerbates disease progression and adds to the challenge of therapeutic development (Poirier et al. 2020). In this context, identifying robust biomarkers and understanding the molecular pathways driving SCLC are essential for advancing the fields of diagnosis, prognosis, and therapy.

Although smoking is still the predominant risk factor for SCLC, emerging evidence suggests that obesity and other modifiable risk factors may also play a role in its pathogenesis (Rudin et al. 2021). Mendelian randomization (MR) analyses, which are less prone to confounding and reverse causation, tend to show a positive association between genetically predicted body mass index (BMI) and SCLC risk, even after adjustment for smoking (Carreras‐Torres et al. 2017; Zhou et al. 2021). Conversely, a number of observational studies have found an inverse association between BMI and lung cancer risk, especially among never‐smokers, in line with the so‐called “obesity paradox” observed in lung cancer (Zhou et al. 2021; Jeong et al. 2019; Zhu and Zhang 2018; Zhao et al. 2022). This obesity paradox in lung cancer may be explained in part by confounding factors such as smoking status and reverse causation, but it is still under active investigation. Central obesity indicators (e.g., waist circumference and waist‐to‐hip ratio) and body composition may actually be more predictive than BMI alone, suggesting that metabolic and body composition factors play a complex role in lung cancer risk (Yu et al. 2018). These findings underscore the importance of investigating metabolic mediators that might be associated with obesity to SCLC risk in the absence of confounding effects. Thus, it is important to separate the direct effect of obesity from indirect effects through smoking or weight loss associated with disease to determine whether the obesity and related metabolic changes are independent risk factor for SCLC.

Obesity‐associated metabolic changes may serve as important mediators in connecting obesity to cancer risk (Avgerinos et al. 2019). As obesity disrupts systemic metabolism, it leads to the production of specific metabolites linked to energy imbalance, lipid metabolism, and inflammation, all of which are increasingly associated with cancer development and progression. Elevated lipid levels, inflammatory markers, and byproducts of lipid oxidation in obese individuals could induce a pro‐tumor microenvironment that supports tumor promotion and development (O'Flanagan et al. 2015). For SCLC, specific metabolites associated with obesity may influence tumor biology (Pedersen et al. 2021; Yu et al. 2021). Metabolites related to lipid and glucose metabolism appear to drive cellular proliferation, immune evasion, and angiogenesis, all of which contribute to SCLC's aggressive progression. Hence, the identification of these obesity‐related metabolites could provide valuable biomarkers and novel therapeutic targets, which could address a critical gap in effective therapy options for SCLC.

MR has emerged as a powerful tool for unraveling complex causal relationships in observational research, by leveraging genetic variants as instrumental variables (IVs) to make causal inferences (Emdin et al. 2017). In order to produce valid causal estimates, MR has three core assumptions: (1) Relevance, which requires that the genetic variants used as IVs are strongly associated with the exposure of interest; (2) Independence, meaning the IVs must be independent of any confounders that are associated with both the exposure and the outcome, ensuring that observed associations are not driven by external factors; and (3) Exclusion Restriction, the genetic variants should affect the outcome solely via their impact on the exposure and not through other routes (Sanderson et al. 2022). Recent developments have expanded this framework to the metabolome‐wide Mendelian randomization (MWMR) framework, enabling systematic evaluation of the causal effects of individual metabolites on disease risk (Duan et al. 2024; Yazdanpanah et al. 2024). This unbiased, hypothesis‐free approach is particularly well‐suited to identifying metabolic mediators, which may uncover the relationship between obesity and the risk of SCLC, thereby providing a novel mechanism by which one or more specific metabolites influence cancer risk.

In this study, we leveraged a MWMR approach to investigate the potential causal role of specific metabolites in mediating the association between obesity and the risk of SCLC. By discerning key metabolites that mediate the link between obesity and SCLC risk, this study sheds light on the metabolic mechanisms contributing to cancer risk and distinguishes direct effects from confounders. These findings could contribute to biomarker development and novel therapeutic strategies, offering targeted approaches for prevention and early intervention in SCLC.

## 2 Method

### 2.1 Study Design

**FIGURE 1:**
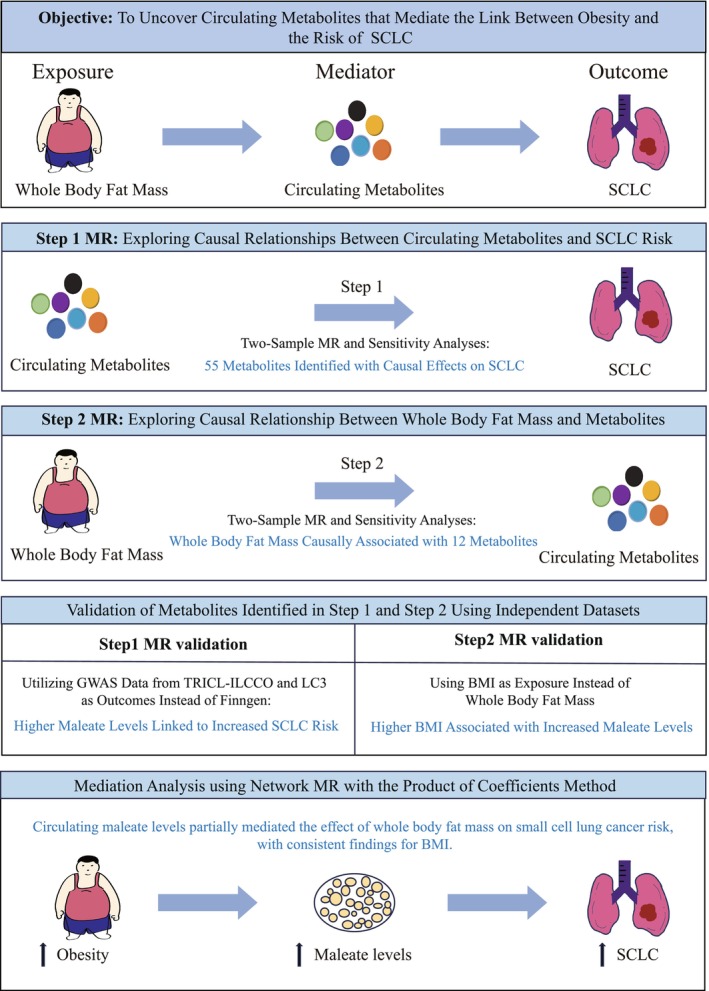
Study overview and analytical strategy.

This study employed the MWMR approach to explore whether specific circulating metabolites mediate the association between obesity and the risk of SCLC (Figure 1). The study was designed to consist of two main MR analysis steps, followed by validation analyses to confirm the robustness of the results. In Step 1, we sought to identify circulating metabolites causally related to SCLC risk. Genetic association data for SCLC were obtained from a GWAS in the Finnish FinnGen study. GWAS for 1400 circulating metabolites were derived from the Canadian Longitudinal Study on Aging (CLSA) cohort, and these metabolites were assessed for their potential causal influence on SCLC risk. Of these metabolites, 77 metabolites were identified by two‐sample MR analyses as showing a significant association with SCLC risk. Sensitivity analyses were performed to confirm the robustness of our findings and to detect potential pleiotropy, and 55 metabolites with significant associations with SCLC risk were identified. In Step 2, we examined whether whole body fat mass has a causal effect on the levels of the 55 metabolites identified in Step 1. Genetic variants associated with whole body fat mass, sourced from the UK Biobank (UKB), served as IVs to assess their effects on the circulating metabolite levels. The purpose of this was to explore whether whole body fat mass has a causal effect on specific metabolites identified in Step 1. Using MR analysis, we found 24 metabolites that were causally affected by whole body fat mass. Further sensitivity analyses, including reverse causation tests, were conducted to further refine the results and this process identified 12 metabolites that were significantly associated with both whole body fat mass and SCLC risk. To replicate the results of Step 1, we performed an additional series of MR analyses based on genetic data for SCLC from a meta‐analysis of GWAS datasets, the Transdisciplinary Research for Cancer in Lung (TRICL) of the International Lung Cancer Consortium (ILCCO) and the Lung Cancer Cohort Consortium (LC3). This validation aimed to confirm the causal effect of maleate, one of the key metabolites identified, on SCLC risk, yielding further support for the mediation effect. For Step 2, we also carried out additional MR analyses substituting BMI as a proxy for whole body fat mass to determine whether it also influences maleate levels. BMI‐associated genetic data were obtained from the Genetic Investigation of Anthropometric Traits (GIANT) consortium. This step of validation was intended to confirm the observed relationship between whole body fat mass and maleate concentration, therefore confirming the findings from Step 2 and strengthening the evidence that the maleate could be a potential mediator of obesity‐related cancer risk. Finally, we conducted a mediation analysis to quantify the proportion of the effect of whole body fat mass or BMI on SCLC risk that is mediated through maleate. We employed the product of coefficients method to estimate the indirect effect of obesity on SCLC risk, which is thought to be mediated through circulating maleate levels. This study was conducted following the Mendelian Randomization STROBE (Strengthening the Reporting of Observational Studies in Epidemiology) checklist to ensure the rigor and transparency of the research process (Skrivankova et al. 2021).

### 2.2 Step 1: Identification of Metabolites Causally Associated With SCLC Risk

#### 2.2.1 Data Source

Metabolite summary statistics were obtained from a dataset that included 1091 metabolites and 309 metabolite ratios, analyzed in 8299 individuals from the CLSA cohort (Chen et al. 2023). Genetic association data for SCLC were sourced from the FinnGen study (R11 version) (Kurki et al. 2023). The dataset comprised 855 SCLC cases and 345,118 controls of European ancestry for a total of 21,304,551 genetic variants. Description of the datasets used in this work is summarized in Table in Table S1.

#### 2.2.2 Instrumental Variable Selection Criteria

To select IVs, we identified single nucleotide polymorphisms (SNPs) that achieved genome‐wide significance (*p* < 1 × 10^−5^) and demonstrated independence (*r* ^2^ < 0.001, with a clumping distance of 10 Mb). Each SNP's robustness as an instrument was assessed through the calculation of the *F*‐statistic. To estimate the proportion of variance explained (*R* ^2^) by each SNP, we employed the formula: $R^{2}=\frac{2\times \beta^{2}\times \mathrm{EAF}\times \left(1-\mathrm{EAF}\right)}{2\times \beta^{2}\times \mathrm{EAF}\times \left(1-\mathrm{EAF}\right)+2\times {\mathrm{SE}}^{2}\times N\times \mathrm{EAF}\times \left(1-\mathrm{EAF}\right)}$, where β is the SNP's effect estimate on the exposure, EAF represents the effect allele frequency, SE is the standard error of the estimate, and N is the sample size for the exposure (Shim et al. 2015). Using the derived *R* ^2^, we then calculated the *F*‐statistic as follows: $F=\frac{R^{2}\times \left(N-2\right)}{1-R^{2}}$ (Palmer et al. 2012). In line with established MR guidelines, we retained only those SNPs with *F*‐statistics greater than 10, in order to assure the quality of strong instrument and hence reducing potential weak instrument bias, which may result in invalid causal inferences (Staiger and Stock 1994). This threshold helped to safeguard the robustness of the MR analysis by minimizing the risk of weak instrument bias.

#### 2.2.3 MR and Sensitivity Analysis

To investigate the causal relationship between circulating metabolites and the risk of SCLC, we conducted a two‐sample MR analysis. The inverse‐variance weighted (IVW) method was employed as the primary approach in the MR analysis, using a random‐effects model. This allows for the potential heterogeneity among genetic instruments by assuming a distribution for the true causal effects, and the method is therefore more robust to the presence of heterogeneous causal estimates across SNPs (Burgess et al. 2013). The IVW method has several advantages: it combines the effect estimates from multiple SNPs into a single causal estimate, which maximizes statistical power and minimizes the probability of false positive findings under the assumption of no horizontal pleiotropy. By integrating information across SNPs, the IVW method effectively captures the overall genetic effect on the outcome, assuming all SNPs meet the IV assumptions. To further check the robustness of our findings and to verify the causal relationships, we performed additional sensitivity analyses using several alternative MR methods, including MR‐Egger, Weighted Median, Simple Mode, and Weighted Mode. These methods provide complementary approaches to dealing with potential complexities, including pleiotropy and heterogeneity of the instrumental variables. A critical point in our refutation test was to check whether the direction of the causal effect (Beta) was similar across methods. The consistency in the direction of the Beta coefficients between the IVW and the alternative approaches is critical to establishing the reliability of our causal inference. If the causal estimates from various methods have consistent direction, this indicates that the association is unlikely driven by confounding factors such as pleiotropy or biases inherent to any specific method. On the contrary, if discrepancies in the Beta direction were observed, this would suggest potential issues concerning the validity of the IVs, suggesting presence of horizontal pleiotropy or other biases. Therefore, we imposed that the Beta directions from the alternative methods be consistent with that obtained from the IVW analysis to ensure the robustness and credibility of our conclusions. To further ensure the validity of our findings, we carried out several sensitivity analyses to identify and adjust for horizontal pleiotropy, where SNPs might influence the outcome through pathways other than the exposure. First, we performed MR‐Egger regression, a robust sensitivity analysis designed to detect and adjust for potential pleiotropic effects (Bowden et al. 2015). MR‐Egger differs from the IVW by estimating an intercept term in the regression model and this intercept captures the presence of directional pleiotropy. This approach yields unbiased causal estimates even when all SNPs exhibit pleiotropic effects, as long as the assumption of independent pleiotropic effects holds; specifically, the genetic associations with the exposure must be uncorrelated with their pleiotropic effects on the outcome (Burgess and Thompson 2017). Second, to evaluate heterogeneity among the SNP‐specific causal estimates, we applied Cochran's *Q* test, which is widely used in MR studies to evaluate the variability of causal estimates by multiple instruments. If the causal estimates are more diverse than what can be expected by chance, it indicates that pleiotropy or other biases may included (Bowden et al. 2017). Cochran's *Q* test thus serves as a formal diagnostic tool to assess the consistency of SNP effect sizes under the IVW model (Greco M et al. 2015). Finally, we conducted a reverse MR analysis to further investigate the potential bidirectional relationship among obesity, circulating metabolites and SCLC risk, thereby ensuring that our findings were not influenced by reverse causality. Taken together, these sensitivity analyses strengthen the robustness of our MR findings, providing more confidence that the observed causal relationship between circulating metabolites and SCLC risk is not confounded by pleiotropic biases or other sources of heterogeneity.

#### 2.2.4 Statistical Analyses

All statistical analyses and visualizations were performed using R statistical software (version 4.4.1). Specifically, we applied the TwoSampleMR package to carry out MR analyses, which has been widely used in MR studies for performing both standard and sensitivity analyses. In all analyses, P‐values < 0.05 were considered statistically significant. For sensitivity analyses, the following assumptions were tested: (i) horizontal pleiotropy, (ii) heterogeneity of causal estimates across instruments, and (iii) reverse causality.

### 2.3 Step 2: Assessing the Causal Effect of Whole Body Fat Mass on Metabolite Concentrations

#### 2.3.1 Data Sources and MR Analysis

The genetic summary statistics for whole body fat mass, used as the exposure in this analysis, were derived from the Neale Lab's GWAS of the UKB round 2, involving 330,762 European‐ancestry participants and covering 10,894,596 genetic variants. The outcome data on metabolites remain consistent with those used in Step 1. IVs were determined with the same selection criteria as in Step 1, but here were screened based on genome‐wide significance at a more stringent threshold (*p* < 5 × 10^−8^) in order to derive robust IVs. Sensitivity and MR analyses in Step 2 followed the same analytical framework, parameters, and assumptions of Step 1, guaranteeing consistent methodology throughout the evaluation.

### 2.4 Step 1 MR Validation

#### 2.4.1 Data Sources and MR Analysis

This stage aimed to confirm the validity of metabolites that were identified in the screening stages as candidate mediators in the relationship between obesity and SCLC risk. For this validation, we utilized genetic summary data for lung cancer outcomes from the TRICL/ILCCO and LC3 consortia (McKay et al. 2017). The TRICL‐ILCCO dataset provided genetic data on 11,177 lung cancer cases and 40,396 controls, while LC3 contributed data from an additional 18,089 lung cancer cases and 16,054 controls, totaling 29,266 lung cancer cases and 56,450 controls for the two consortia. This GWAS meta‐analysis included participants of European ancestry genotyped on multiple platforms, with imputation performed based on the 1000 Genomes Project to improve data quality and facilitate cross‐study harmonization. The meta‐analysis enabled stratification by lung cancer histological subtypes, which enabled us to perform targeted analyses focused specifically on SCLC among other subtypes. This dataset contains 2664 cases of SCLC, providing a robust sample size for examining potential causal links between obesity‐associated metabolites and SCLC risk. To ensure methodological consistency and rigor, the selection criteria for IVs, MR analytical methods, and sensitivity analyses were identical to those employed in Step 1.

### 2.5 Step 2 MR Validation

#### 2.5.1 Data Sources and MR Analysis

We performed an additional MR analysis as a validation using BMI, a widely accepted proxy for whole body fat mass, to determine its causal effect on those previously identified metabolites associated with SCLC risk. The GWAS summary statistics for BMI were obtained from the GIANT consortium, which includes a large‐scale meta‐analysis involving 681,275 individuals of European ancestry and covering over 2.5 million genetic variants (Locke et al. 2015). This dataset provided a robust basis for estimating the genetic influences of BMI on circulating metabolite levels. The criteria for selecting IVs, along with the MR analysis and sensitivity assessment methods, were applied in alignment with those established in Step 2 to ensure methodological consistency.

### 2.6 Mediation Analysis

To evaluate the mediating effect of circulating maleate in the relationship between obesity and SCLC risk, the product of coefficients method was applied to two exposures: whole body fat mass and BMI. The first step estimated the causal effect of the exposure on the mediator (β_EM) and the second step evaluated the causal effect of the mediator on the outcome (β_MO). The total effect of the exposure on the outcome (β_EO) was then calculated to quantify the overall impact of obesity on SCLC risk, including both direct and mediated effects. Finally, the mediation proportion was determined using the product‐of‐coefficients method (Mediation Proportion = $\frac{\beta \_\mathrm{EM}\times \beta \_\mathrm{MO}}{\beta \_\mathrm{EO}}$), providing a quantitative assessment of the extent to which maleate mediates the relationship between obesity and SCLC risk. This approach enabled a detailed examination of both direct and indirect causal pathways, highlighting maleate's role as a potential metabolic mediator in obesity‐related SCLC risk.

## 3 Result

### 3.1 Step1: Identification of Metabolites Associated With SCLC Risk

**FIGURE 2:**
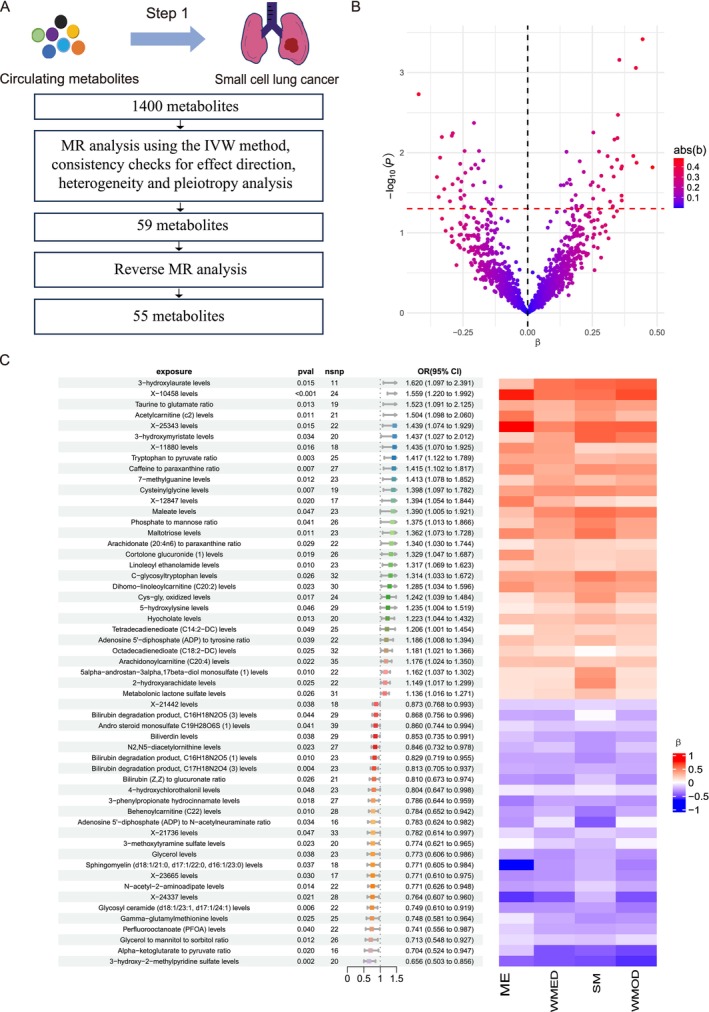
Causal effects of circulating metabolites on SCLC risk. (A) Step 1 analytical workflow. (B) The volcano plot visualizes the association between metabolites and SCLC risk, plotting ‐log10‐transformed *p*‐values (*y*‐axis) against effect estimates (*β*‐values, *x*‐axis). The dashed red line indicates the significance threshold (*p* = 0.05). (C) The forest plot summarizes the IVW results for the metabolites significantly associated with SCLC risk. Each metabolite is presented with its odds ratio (OR), 95% confidence interval (CI), and corresponding *p*‐value, alongside the number of SNPs (*n*SNPs) supporting the analysis. Adjacent to the forest plot, a heatmap displays the effect estimates (*β*‐values) derived from additional MR methods, including MR‐Egger (ME), weighted median (WMED), simple mode (SM), and weighted mode (WMOD). The heatmap's color scale reflects the magnitude and direction of effect sizes, with red indicating positive associations and blue denoting negative associations.

In Step 1, we performed a two‐sample MR study to assess the causal relationship between 1400 circulating metabolites and SCLC risk, using the IVW method as the primary approach (Figure 2A; Table S2). At a significance threshold of *p* < 0.05, we identified 77 metabolites that were genetically associated with SCLC risk (Figure 2B; Table S3). To ensure the robustness and validity of these findings, we applied a series of stringent criteria and several sensitivity analyses. We firstly checked the consistency across five different MR methods: IVW, MR‐Egger, weighted median, simple mode, and weighted mode. Only those metabolites were retained if their Beta values were consistently greater than zero or less than zero across all methods, thus narrowing down the list to 61 metabolites. Then, potential biases, such as horizontal pleiotropy and heterogeneity were examined. Horizontal pleiotropy, where genetic variants influence the outcome through pathways unrelated to the exposure, was assessed using MR‐Egger regression. In this approach, the intercept term represents a formal test for the presence of directional pleiotropy, and the slope is a causal estimate under correction for such pleiotropic effects. Two metabolites were excluded at this step due to significant evidence of pleiotropy (Table S3). We also performed Cochran's *Q* test for the presence of heterogeneity among causal estimates across IVs. The remaining 59 metabolites showed no evidence of significant heterogeneity, suggesting that the observed associations were unlikely influenced by bias or outliers (Table S3). To further strengthen causal inference and rule out reverse causality, reverse MR analysis was performed. Here, genetic variants associated with SCLC were used as IVs to test whether SCLC could influence the levels of the identified metabolites. Four metabolites showed significant evidence of reverse causality, indicating they were likely downstream effects of the disease rather than upstream mediators (Table S4). Metabolites exhibiting evidence of reverse causality were excluded, thus 55 metabolites were left with robust and consistent causal associations with SCLC risk (Figure 2C).

### 3.2 Step 2: Identification of Circulating Metabolites Influenced by Whole Body Fat Mass

**FIGURE 3:**
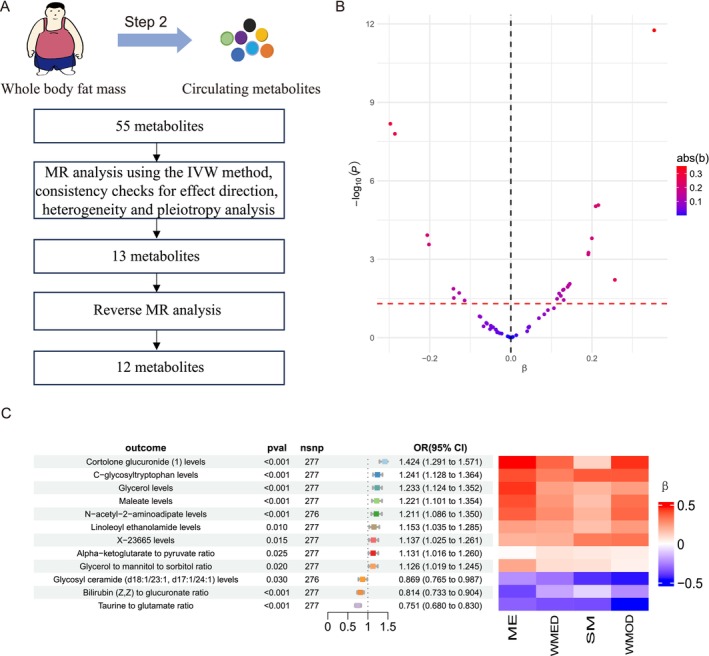
Causal relationship between whole body fat mass and metabolite levels. (A) Step 2 analytical workflow. (B) The volcano plot illustrates the MR results for these metabolites, with the *x*‐axis representing the effect size (β) and the *y*‐axis showing −log10 (*p*‐value). Each point corresponds to a metabolite, colored according to the absolute magnitude of β (abs(β)); larger effects are highlighted in red. (C) The forest plot presents the IVW MR estimates for the 12 metabolites, including their odds ratios (ORs) with 95% confidence intervals (CIs). Adjacent to the forest plot, a heatmap displays *β*‐values derived from four supplementary MR methods: ME, WMED, SM, and WMOD.

In Step 2, we further investigated whether whole body fat mass (as a proxy for obesity) has causal effect on the 55 metabolites identified in Step 1. We conducted a MR analysis, using whole body fat mass as the exposure and the 55 metabolites as the outcomes, with the IVW method as the primary approach (Figure 3A; Table S5). MR analysis identified 24 metabolites causally affected by whole body fat mass (Figure 3B; Table S6). To confirm the strength of these associations, we conducted a series of sensitivity analyses. First, metabolites that displayed inconsistent beta directions across multiple MR methods were excluded, resulting in 16 metabolites for the subsequent analysis (Table S6). Next, three circulating metabolites were excluded due to significant heterogeneity (Table S6). Reverse MR analysis was then performed to determine the potential for reverse causality, which led to the removal of 1 circulating metabolite (Table S7). Following the above process, a total of 12 metabolites were retained, exhibiting robust and consistent associations with whole body fat mass. Among the 12 identified metabolites, genetically predicted increases in whole‐body fat mass were significantly associated with higher levels of Cortolone glucuronide, C‐glycosyltryptophan, Glycerol, N‐acetyl‐2‐aminoadipate, Linoleoyl ethanolamide, X‐23665, alpha‐ketoglutarate to pyruvate ratio, glycerol to mannitol to sorbitol ratio, and Maleate. Conversely, genetically predicted higher whole‐body fat mass was associated with lower levels of Glycosyl ceramide, Bilirubin (Z,Z) to glucuronate ratio, and Taurine to glutamate ratio (Figure 3C). These findings highlight a subset of metabolites whose levels are robustly influenced by whole‐body fat mass, providing candidate mediators for obesity‐related SCLC risk.

### 3.3 Validation of Step 1 Findings Using TRICL‐ILCCO and LC3 Data

To validate the results of Step 1, we used independent SCLC data from the TRICL‐ILCCO and LC3 consortia to assess whether the metabolites identified in Step 2 were causally associated with the risk of SCLC. In this follow‐up analysis, we used the 12 metabolites retained from Step 2 as exposures and performed MR with SCLC risk as the outcome (Table S8). After applying a significance threshold of *p* < 0.05, only maleate remained significantly associated with SCLC risk. Further analysis indicated that maleate also met the criteria for consistency in effect direction across five MR methods and showed no evidence of heterogeneity or pleiotropy. Finally, reverse MR analysis ruled out reverse causality, suggesting that the observed association was likely driven by maleate's influence on SCLC risk rather than the reverse (Table S9). These results provide evidence that maleate may mediate the association between obesity and SCLC risk to some extent.

### 3.4 Validation of Step 2 Findings Using BMI as a Proxy for Whole Body Fat Mass

To further validate the results from Step 2, we performed a MR analysis with BMI as the exposure and maleate levels as the outcome, substituting the whole body fat mass with BMI. BMI is a widely used proxy for whole body fat mass and offers a more accessible measure for large‐scale genetic studies, making it an appropriate alternative for validating our findings. In this analysis, we applied the IVW method to estimate the causal effect of BMI on maleate levels. The MR results revealed genetically higher BMI was associated with increased maleate levels (Beta = 0.154, SE = 0.051, *p* = 0.0024) (Table S10). Importantly, this association met the principal validation criteria, with consistent directions of effects across five MR methods (IVW, MR‐Egger, weighted median, simple mode, and weighted mode), indicating robustness to variation in methodology. In addition, sensitivity analyses revealed no evidence of heterogeneity, as assessed by Cochran's *Q* test, or directional pleiotropy, based on MR‐Egger regression. In order to exclude the possibility of reverse causality, we performed a reverse MR analysis using maleate levels as the exposure and BMI as the outcome. This analysis found no significant evidence that maleate influenced BMI, supporting the unidirectional nature of the causal relationship (Table S11). These results strengthen the evidence for maleate being a metabolite causally influenced by obesity (measured by BMI) and suggest that it may act as a mediator in the association between obesity and risk of SCLC.

### 3.5 Mediation Analysis of Maleate in the Association Between Obesity and SCLC Risk

To assess the extent to which circulating maleate mediates the relationship between obesity and SCLC risk, we conducted a mediation analysis using MR with two sets of datasets: whole body fat mass with SCLC from FinnGen and BMI with SCLC from the TRICL‐ILCCO and LC3 consortia.

**FIGURE 4:**
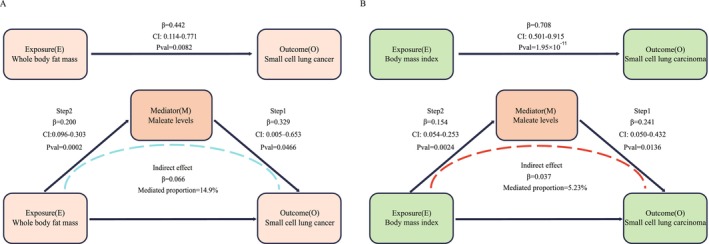
Maleate levels as a mediator in the causal pathway from obesity to SCLC risk. (A) Direct pathway: The top arrow indicates the direct effect of whole body fat mass (Exposure, E) on SCLC risk (Outcome, O), with an effect size of BetaEO = 0.442, CI: 0.114–0.771, and a *p*‐value of 0.0082. Mediation analysis: The central part of the diagram highlights the role of maleate levels as a mediator (Mediator, M). The arrow from whole body fat mass to maleate levels shows the association identified in Step 2 MR (BetaEM = 0.200, CI: 0.096–0.303, *p* = 0.0002). The arrow from maleate levels to SCLC risk shows the result of Step 1 MR (BetaMO = 0.329, CI: 0.005–0.653, *p* = 0.0466). Indirect effect: The dashed arrow represents the indirect effect of whole body fat mass on SCLC risk through maleate levels, with a calculated indirect effect of BetaEO = 0.066. The proportion of the effect mediated by maleate is 14.9%. (B) Direct pathway: The top arrow indicates the direct effect of BMI (Exposure, E) on SCLC risk (Outcome, O), with an effect size of BetaEO = 0.708, CI: 0.501–0.915, and a *p*‐value of 1.95 × 10^−11^. Mediation analysis: The central part of the diagram highlights the role of maleate levels as a mediator. The arrow from BMI to maleate levels shows the association identified in Step 2 MR (BetaEM = 0.154, CI: 0.054–0.253, *p* = 0.0024). The arrow from maleate levels to SCLC risk shows the result of Step 1 MR (BetaMO = 0.241, CI: 0.050–0.432, *p* = 0.0136). Indirect effect: The dashed arrow represents the indirect effect of BMI on SCLC risk through maleate levels, with a calculated indirect effect of BetaEO = 0.037. The proportion of the effect mediated by maleate is 5.23%.

To quantify the extent to which circulating maleate mediates the effect of whole‐body fat mass on SCLC risk, we performed a MR mediation analysis using the product‐of‐coefficients method. First, using the IVW method, we found a significant positive association between whole body fat mass and maleate levels (*β* = 0.200, 95% CI: 0.096–0.303, *p* = 0.0002) (Figure 4A; Table S12). Second, we assessed the effect of circulating maleate levels on SCLC risk. The MR analysis revealed that genetically predicted higher maleate levels were associated with an increased risk of SCLC (*β* = 0.329, 95% CI: 0.005–0.653, *p* = 0.0466). We then estimated the total effect of whole body fat mass on SCLC risk, which confirmed that increased whole body fat mass is positively associated with a higher risk of SCLC (*β* = 0.442, 95% CI: 0.114–0.771, *p* = 0.0082). To validate the robustness of our results, we performed sensitivity analyses that showed no evidence of heterogeneity, horizontal pleiotropy, or reverse causality, reinforcing the reliability of our causal inferences. Our mediation analysis revealed that circulating maleate levels partially mediate the relationship between whole body fat mass and SCLC risk, with a mediation proportion of 14.9% (95% CI: −1.68%, 31.4%).

In the second analysis, BMI was used as the exposure, with SCLC outcomes obtained from the TRICL‐ILCCO and LC3 consortia. The IVW analysis showed a significant positive association between BMI and circulating maleate levels (*β* = 0.154, 95% CI: 0.054–0.253, *p* = 0.0024) (Figure 4B; Table S13). Similarly, maleate levels were causally linked to an increased risk of SCLC (*β* = 0.241, 95% CI: 0.050–0.432, *p* = 0.0136). The total effect of BMI on SCLC risk was robust, with BMI associated with increased SCLC risk (*β* = 0.708, 95% CI: 0.501–0.915, *p* = 1.95 × 10^−11^). Mediation analysis revealed that maleate mediates 5.23% (95% CI: −0.125%, 10.6%) of the effect of BMI on SCLC risk. Together, these results indicate that maleate partially mediates the causal relationship between obesity and SCLC risk.

## 4 Discussion

In this study, we used MR analysis to assess the causal role of circulating metabolites in mediating the relationship between obesity and the risk of SCLC. In particular, our MR analysis identified maleate levels as a potential mediator in the causal pathway between obesity and SCLC risk, accounting for a proportion of the total effect. These findings underscore the importance of metabolic intermediates in the pathophysiology of obesity‐related cancers and introduce a novel layer to the understanding by which obesity may increase cancer risk.

Several observational studies have reported associations between BMI and the risk of lung cancer (Yu et al. 2018; Dewi et al. 2016). Remarkably, some studies have reported that higher BMI may be linked to a decreased risk of lung cancer even after adjusting for known confounders, such as smoking and other lifestyle factors (Sanikini et al. 2018; Duan et al. 2015). Nevertheless, these studies are at risk of residual confounding where factors such as diet, physical activity, or genetic predisposition could be influencing the true relationship between obesity and cancer risk. In addition, observational studies cannot easily address reverse causality, in which the disease itself could lead to weight loss, rather than obesity causing the disease. In contrast, MR provides a powerful tool to address these concerns. MR leverages genetic variants as IVs to estimate causal relationships between exposures (e.g., obesity) and outcomes (e.g., cancer). Consistent with the previous findings, Zhou et al. applied MR to examine the effect of BMI on lung cancer risk, including SCLC, and found that BMI was still positively associated with SCLC risk, even after adjusting for smoking behaviors (Zhou et al. 2021). This is in line with our results, where we demonstrate that obesity, as measured by whole body fat mass or BMI, represents a risk factor for SCLC. The application of MR emphasizes its ability to provide more convincing estimates of causality, reducing the impact of confounding factors and reverse causality that commonly affect traditional observational studies.

SCLC is a highly aggressive malignancy often diagnosed at advanced stages due to non‐specific symptoms, limiting treatment options and contributing to poor survival rates (Tsoukalas et al. 2018). Metabolomics, an emerging systems biology tool that enables the comprehensive characterization of small‐molecule metabolites in biological samples, is promising for revealing the molecular mechanism of SCLC (Derveaux et al. 2018). As intermediates and end products of cellular metabolism, metabolites have been reported to be involved in cancer processes such as differentiation, proliferation, and DNA repair, and thus can be utilized as potential biomarkers for diagnosis and therapeutic targets (Hanahan and Weinberg 2011). An analysis of 30 serum samples from SCLC patients and 25 healthy controls based on nuclear magnetic resonance spectroscopy demonstrated notable metabolic alterations in SCLC patients (Pedersen et al. 2021). In the SCLC patients, several metabolite changes were identified such as increased levels of succinate, glutamic acid, 3‐hydroxybutyric acid, and acetone, and decreased levels of amino acids including leucine, isoleucine, and valine. These metabolic disruptions involve pathways related to the tricarboxylic acid cycle, lipid metabolism, amino acids, and ketone body metabolism. In this study, we identified two categories of metabolites: those associated with obesity‐related metabolic alterations and those with a causal effect on the risk of SCLC. The discovery of obesity‐associated metabolites offers novel insights into the metabolic pathways through which obesity may increase SCLC risk, indicating systemic metabolic alterations that could influence tumor development. Meanwhile, the metabolites associated with SCLC risk highlight specific metabolic vulnerabilities that may serve as potential biomarkers for early diagnosis or therapeutic targets for the disease. Together, these findings are consistent with existing experimental research and provide novel avenues for targeting metabolites as potential biomarkers or therapeutic targets in the prevention and treatment of obesity and SCLC.

Maleate, a dicarboxylic acid metabolite, is a well‐established experimental model compound, primarily known for its selective impact on renal proximal tubular cells, causing subsequent disruption of kidney function (Zager et al. 2008). Mechanistically, maleate enters proximal tubular cells via organic anion transport systems and interacts with succinyl‐CoA: 3‐ketoacid CoA transferase, forming a stable CoA derivative that depletes cellular CoA pools (Pacanis et al. 1981). This exhaustion reduces the activation and metabolism of fatty acids and results in a direct reduction in ATP production (Kellerman 1993). Additionally, maleate interacts with the thiol groups of glutathione, inducing oxidative stress and compromising cellular defense mechanisms (MimiĆ‐Oka and SimiĆ 1997; Nissim and Weinberg 1996; Tirmenstein et al. 2000). Maleate also inhibits Na^+^‐K^+^‐ATPase and H^+^‐ATPase activities in proximal tubules, significantly reducing bicarbonate, phosphate, and protein reabsorption (Zager 2000). This leads to bicarbonate back‐diffusion into the tubular lumen, exacerbating metabolic acidosis. Furthermore, maleate stimulates glutamine metabolism, resulting in metabolic blockade at α‐ketoglutarate dehydrogenase and disrupting mitochondrial NADH production (Gougoux et al. 1985). By mimicking hypoxia‐ and ATP depletion‐associated tubular injury, maleate has proven to be an invaluable experimental tool for studying nephrotoxicity, proximal tubule‐specific dysfunctions, and potential therapeutic strategies such as antioxidant interventions with compounds like curcumin.

Most of the studies on maleate have primarily focused on animal models, and human‐centered studies are still quite limited. Notably, recent studies indicate that maleate is also involved in various systemic metabolic processes. Elevated maleate levels in individuals with type 2 diabetes mellitus have been associated with disrupted central carbon metabolism, such as alterations in glycolysis, gluconeogenesis, and the tricarboxylic acid cycle (Shu et al. 2024). Interestingly, metformin, a first‐line treatment for T2DM, has been shown to significantly lower circulating maleate levels along with the recovery of metabolic homeostasis. These findings highlight the maleate as a dynamic biomarker of metabolic stress and suggest the feasibility of monitoring metabolic derangement using maleate in clinical practice. In addition, the toxicogenomic profile of maleate further implicates it as a cancer related molecule (Lin et al. 2014). In silico studies have shown maleate as a key player in various disease categories, including cardiovascular, neurological, and neoplastic disorders, indicating its broad biological significance. Mechanistically, the potential tumorigenic contribution of maleate may be mediated by interference with mitochondrial function and cellular energy metabolism, both of which are crucial processes in the development of obesity and cancer. Its upregulation in obesity‐related metabolic states may exacerbate oxidative stress and energy deficits, particularly in lung epithelial cells, thereby promoting a microenvironment conducive to the initiation and progression of SCLC. The hypothesis that maleate could be the link between obesity and SCLC needs to be confirmed by further experiments. Particularly, studies employing models that more closely mimic the metabolic and oncogenic milieu associated with obesity‐related cancers are essential to elucidate the exact function of maleate in cancer initiation and progression.

This study has several advantages. MR analysis provides a robust framework for causal inference and limits the influence of confounding and reverse causality. The multi‐step design, utilizing large and diverse biobank data, reinforces the findings and offers robust evidence. Sensitivity analyses guarantee the reliability of the results. The identification of maleate as a mediator between obesity and SCLC risk is a novel contribution, potentially informing targeted interventions. Further validation in independent datasets also confirms the robustness of the findings, which could pave the way for novel therapeutic strategies for reducing obesity‐associated cancer risks.

Despite these strengths, this study has several limitations. First, it is derived from European populations, which may limit the generalizability of our findings to other ethnic groups. The role of maleate as a mediator between obesity and SCLC risk requires further validation in non‐European populations. Second, the use of genetic proxies for metabolite concentrations, while providing a strong foundation for causal inference, may not fully account for the dynamic and tissue‐specific aspects of maleate metabolism that might differ between tissues and environmental conditions. Third, we did not perform multiple testing correction in the MR analyses. However, the consistent causal direction observed across five complementary MR methods, together with replication in independent datasets, enhances the credibility of our findings. Finally, while the mediation analysis indicated a potential mediating role of circulating maleate in the relationship between obesity and SCLC risk, the confidence interval for the estimated mediation proportion included the null, reflecting statistical uncertainty. These results, therefore, should be interpreted with caution and warrant further validation.

## 5 Conclusion

MR analysis identifies maleate as a potential metabolic link between obesity and SCLC risk, which provides valuable insights into the manner by which obesity‐induced metabolic alterations may contribute to the pathogenesis of SCLC. This finding highlights the importance of metabolic pathways in understanding the relationship between obesity and cancer and suggests that targeting maleate or its related pathways could open novel avenues for therapeutic interventions.

## Author Contributions

**Kui Li:** investigation (equal), writing – original draft (equal). **Zhaodi Yang:** investigation (equal), writing – original draft (equal). **Haifei Li:** investigation (equal), writing – original draft (equal). **Cheng Yan:** designed the study and reviewed the final manuscript.

## Ethics Statement

The authors have nothing to report.

## Conflicts of Interest

The authors declare no conflicts of interest.

## Supporting information


**Table S1:** Details of traits used in the MR analysis.
**Table S2:** Instrumental variables of metabolites and harmonization results in the step 1 MR.
**Table S3:** MR results for the effect of metabolites on small cell lung cancer.
**Table S4:** Reverse MR result for the effects of small cell lung cancer on metabolites.
**Table S5:** Instrumental variables of exposure and harmonization results in the step 2 MR.
**Table S6:** MR results for the effect of whole body fat mass on metabolite levels.
**Table S7:** Reverse MR result for the effects of metabolites on whole body fat mass.
**Table S8:** MR results for the effect of 12 metabolites on small cell lung carcinoma data (validation analyses for the step 1 MR).
**Table S9:** Reverse MR result for the effects of small cell lung carcinoma risk on maleate levels.
**Table S10:** MR analyses for the effect of body mass index on metabolite maleate levels (validation analyses for the step 2 MR).
**Table S11:** Reverse MR result for the effects of maleate levels on body mass index.
**Table S12:** The impact of maleate levels as a mediator in the association between whole body fat mass and small cell lung cancer.
**Table S13:** The impact of maleate levels as a mediator in the association between BMI and small cell lung cancer.

## Data Availability

All data used in this study is available in publicly accessible databases. For further inquiries, please contact the corresponding authors.
